# Partisan styles of self-presentation in U.S. Twitter bios

**DOI:** 10.1038/s41598-023-50810-0

**Published:** 2024-01-11

**Authors:** Liam Essig, Daniel DellaPosta

**Affiliations:** 1 Department of Sociology and Criminology, Pennsylvania State University, University Park, PA 16802 USA; 2 Center for Social Data Analytics, Pennsylvania State University, University Park, PA 16802 USA

**Keywords:** Psychology, Human behaviour, Computational science, Information technology

## Abstract

Political polarization in the United States goes beyond divided opinions on key political issues, extending to realms of culture, lifestyle, and social identity once thought to be apolitical. Using a sample of 1 million Twitter bios, this study investigates how users’ partisan self-presentation on social media tends to include cultural as well as political markers. Representing the text in Twitter bios as semantic networks, the study reveals clear partisan differences in how users describe themselves, even on topics that seem apolitical. Consequently, active Twitter users’ political alignments can be statistically inferred from the non-political references in their bios, even in the absence of explicitly partisan language. These findings offer further evidence of partisan polarization that is aligned with lifestyle preferences. Further research is needed to determine if users are aware of that alignment, which might indicate the politicization of lifestyle preferences. The findings also suggest an under-recognized way social media can promote polarization, not through political discourse or argument, but simply in how users present cultural and lifestyle preferences on those platforms.

## Introduction

*“This is the American identity crisis. Not that we have partisan identities, we’ve always had those. The crises emerge when partisan identities fall into alignment with other social identities, stoking our intolerance of each other to levels that are unsupported by our degrees of political disagreement.” * - Lilliana Mason (2018, Pg. 63).

Rising political polarization in the United States has become a central concern in recent years for social scientists and laypeople alike^1–11^. Increasingly, political divisions are reflected not just in the differing opinions that liberals and conservatives hold on key political issues, but also in seemingly apolitical cultural and lifestyle preferences—ranging from musical taste to the consumption of science^12–18^. Speaking to these extensive partisan divides, scholars have argued that much of rising polarization—both in terms of representative population trends and popular perception—is a matter not just of attitudes and opinions (what we believe) but of *social identity* (who we are). Simply put, partisan affiliation is increasingly implicated in peoples’ sense of self^19^, triggering positive feelings toward other members of the partisan in-group and intense negative feelings toward the partisan out-group^20,21^.

The broadening of partisan divisions to incorporate an ever-wider array of previously apolitical topics lends a strong degree of truth to partisan stereotypes of “latte liberals” and “country music conservatives”^12,13,22^. Social and cultural identities that once cut across one another now increasingly layer on top of one another^19^. As a result, people share much in common with their co-partisans (political views, hobbies, media preferences, religious views, etc.) while sharing very little with opposite-partisans. This “layering” of identities exacerbates political conflict and reduces the common ground between opposing sides^23^. Individuals with cross-cutting social identities are more tolerant, less biased, and hold more positive views toward out-groups, while those whose identities are all aligned are more likely to be intolerant, biased, and feel negatively toward out-groups^24–26^.

In this study, we apply natural language processing and semantic network analysis to Twitter bios to further investigate *how* social and cultural identities are polarized along partisan lines. Previous research examining political polarization on Twitter has focused on the content of tweets or patterns of interaction, such as following, re-tweeting, liking, or mentioning other users^18,27,28^. These studies are highly informative regarding personal opinions, attitudes, and the formation of online communities. To focus on the polarization of social identity, however, Twitter bios are comparatively advantageous in that they capture more than just ephemeral utterances; the language in a user’s bio reflects their chosen *self-presentation*, or how they want others to see them^29–31^. The difference between tweets and bios is analogous to the difference between weather and climate. Like the weather, the sentiments expressed in tweets are fleeting and can vary hour by hour or day by day. The climate represents a more consistent underlying force that influences weather patterns. Similarly, Twitter bios tend to be fairly consistent and stable over time, with the average Twitter user tweeting about 12 times a year but changing their bio less than once per year^29^. This stability means that instead of just responding to fleeting events, Twitter bios are likelier to offer a window into the identities people choose to present to the outside world.

Survey research maintains distinct advantages for studying the polarization of public *opinion*. Namely, survey methods can ensure representative samples, consistently measure demographic attributes that are harder to identify on social media, and elicit respondents’ views on any topic chosen by the researcher. However, while surveys remain the gold standard for capturing what people are thinking, they are notably weaker at capturing how people present themselves to others in public settings, where peoples’ opinions are not simply “worn on their sleeves”^32^. Yet, understanding the “political selves” that people present in public settings is critical to understanding how everyday interactions can be polarizing^10,12,33^.

Twitter bios—which simply prompt users to “describe [them]self”—are well suited to this research task. Like other “digital traces”^34^ from online platforms, ranging from Amazon book purchases^17^ to Twitter interactions^28,35–37^, these data are recorded in a natural environment without imposed limitations on topics or content. Still, using digital traces data to examine self-presentation and other social phenomena comes at a price. While these data are rich in information, they lack several strengths of traditional quantitative survey methods, such as representativeness and reliable measurements of demographic attributes. Consequently, while our aim is to describe the politicization of self-presentation in Twitter bios, we will not aim to causally test what accounts for the partisan differences we describe.

we analyze data from a sample of 1 million active-user Twitter bios collected through the Twitter API from 1% of all publicly-available tweets in August 2019. We only include Twitter bios of users with a U.S. time zone as their time setting and their interface language set to English. The text data from the bios were cleaned using standard text cleaning procedures (see “Methods” section for more information). We transform the text into a semantic co-occurrence network where two words are closely linked to the extent that similar users invoke them. We then zero in on the partisan connotations of the words that people use to identify and present themselves to others, using a network-distance metric to capture each word’s partisan lean by comparing its network position to that of a pre-selected set of explicitly partisan terms. By way of preview, we find that even seemingly apolitical identity words—like “mom” or “veteran”—can serve as implicit partisan signals. Finally, we turn our focus from a network of words linked by co-occurrence in bios to the network of users linked by shared word use. To test the strength of partisan differences in “non-political” self-presentation, we examine how accurately we can re-produce partisan linguistic divisions when explicitly partisan words are removed from the bios. To a stunning degree, we find that co-occurrence of apolitical identity words in Twitter bios is sufficient to re-create clusters of active Twitter users who also employ the same partisan identities.

**Figure 1:**
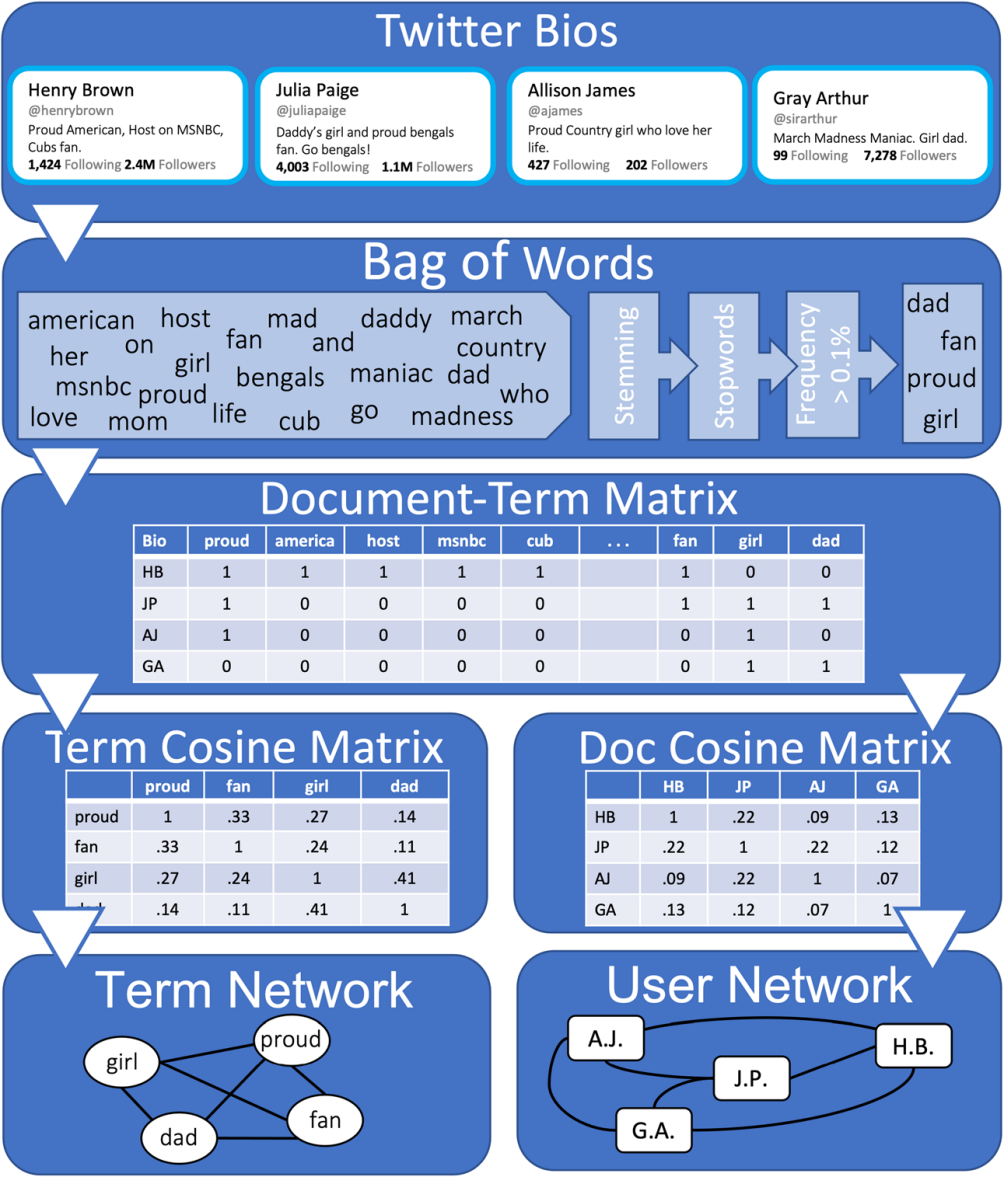
Turning text from Twitter bios into semantic networks. First, text from Twitter bios underwent standard text cleaning procedures, including removing stopwords, stemming words to their roots, and removing infrequent terms. Second, a document term matrix (DTM) was created, with each row representing a Twitter bio and columns representing each word, and then was transformed using *term frequency-inverse document frequency* (TF-IDF) weighting. Third, this matrix was then used to produce cosine-similarity matrices for terms and bios (users). Lastly, both the term and document cosine matrices were projected as weighted networks.

## Results

Figure 1 demonstrates how the raw text in users’ Twitter bios is used to induce semantic networks both for co-occurring terms and for Twitter users themselves. After initially removing empty bios, we apply standard text cleaning procedures to the 811,436 remaining bios and aggregate explicitly partisan terms (e.g., “democrat,” “liberal,” “republican,” “conservative,” “maga”) into single “liberal” and “conservative” labels (see “Methods” Section for more detail). We then create a document-term matrix (DTM) of bios and the words used therein. Infrequently used terms were removed from the document-term matrix, such that only terms that occurred in at least .1% of all bios were included in the analyses. After removing infrequently used terms, some bios were left empty or only had 1 word; these documents were also removed, providing us with a final analytic sample of 677,132 Twitter bios. We created the term network by weighting the cells in the document-term matrix by *term frequency-inverse document frequency* (TF-IDF), which accounts for both the distinctiveness of individual words and the number of words used in each bio. The weighted document-term matrix is then transformed into two separate semantic matrices. The first matrix is a word-by-word cosine similarity matrix that is then represented as a network structure where the nodes represent words and the edges between nodes represent their semantic similarity. The second matrix is a document-by-document (i.e., user-by-user) cosine similarity matrix that is then projected as a network structure where the nodes represent Twitter users and the edges between nodes represent the degree of semantic similarity between two Twitter bios.

**Figure 2:**
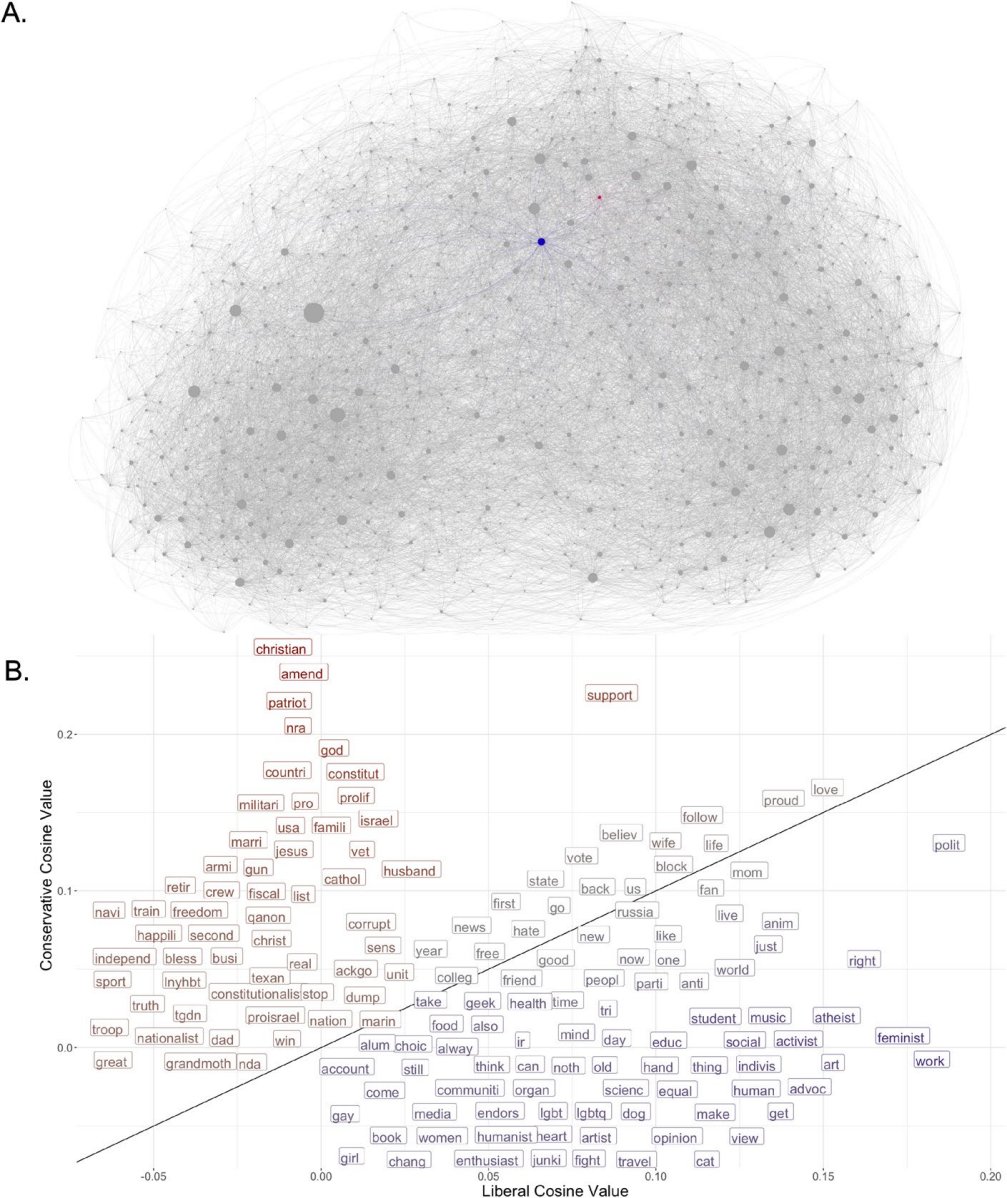
Partisan lean of bio words. Panel (**A**) shows the term co-occurrence network for the most used terms in Twitter bios, with nodes sized by degree (weighted sum of similarities to other terms). The “conservative” and “liberal” nodes are red and blue, respectively. Panel (**B**) plots terms using both liberal and conservative cosine scores for terms with the highest political relevance. Results show that Christian religiosity, American patriotism, and male-specific family words all feature prominently among the most consistently conservative terms in Twitter bios. Conversely, liberal terms are more likely to reference social engagement, intellectualism, and arts/media references.

In Fig. 2, Panel A depicts the term network of semantically related words using force-directed graph embeddings via the Fruchterman-Reingold algorithm^38^, with the blue and red nodes representing aggregations of the terms that we identified as explicitly partisan (blue for liberal/Democratic terms and red for conservative/Republican terms; see “Methods” Section for more detail). The close proximity of the two partisan nodes reflects that most active Twitter users do not explicitly invoke partisan labels in their bios; instead, these terms are generally invoked by a small subset of users who share other terms in common. Additionally, there is a notable difference in size (which reflects network degree, here meaning the weighted sum of connections to other terms) between the two partisan nodes, with the liberal node being tied to more terms. This suggests that those who use liberal identifiers have more diverse language use in their bios, relative to those who use conservative identifiers.

The fact that partisan polarization extends beyond the realm of explicitly political issues, also incorporating diverse cultural and lifestyle signifiers, implies that users may signal their political identities even without invoking explicitly partisan labels in their bios. To investigate this possibility, we measure the partisan lean of the common words people use as part of their self-presentation in bios. To measure the partisan alignment of seemingly non-partisan words, we find the cosine similarity between each word and the aggregated liberal (blue) and conservative (red) nodes, using these similarities to create a *liberal cosine score* and a *conservative cosine score*, respectively, for each term in the network.

We find that seemingly apolitical terms signaling users’ social identities often overlap with explicitly partisan terms in ways that align with existing cultural stereotypes. Panel B of Fig. 2 plots terms with the highest partisan scores along liberal and conservative axes. Results show that Christian religiosity, American patriotism, and male-specific family words all feature prominently among the most consistent signals of conservative political identity in Twitter bios, with “christian,” “patriot,” and “husband” among the most conservative. For liberals, the arts, intellectualism, and social/community orientation featured most prominently, with “music,” “feminist,” “activist,” and “equal” being among the more consistent signals of liberal political identity.

Finally, we turn our attention from bio terms linked by co-occurrence to active Twitter users linked by shared term usage in bios. We have seen that even many bio terms that are not explicitly political can nonetheless serve as implicit signals of users’ partisan identities. However, are such signals strong enough that we could accurately identify partisanship using *only* non-political terms in users’ bios? To the extent that the use of identity signifiers maps closely onto partisanship, liberal and conservative users should tend to almost exclusively use terms that code implicitly as, respectively, liberal or conservative. In a term co-usage network, liberals and conservatives should still fall into distinct linguistic clusters even when we *remove* the explicitly partisan terms from their bios.

**Figure 3:**
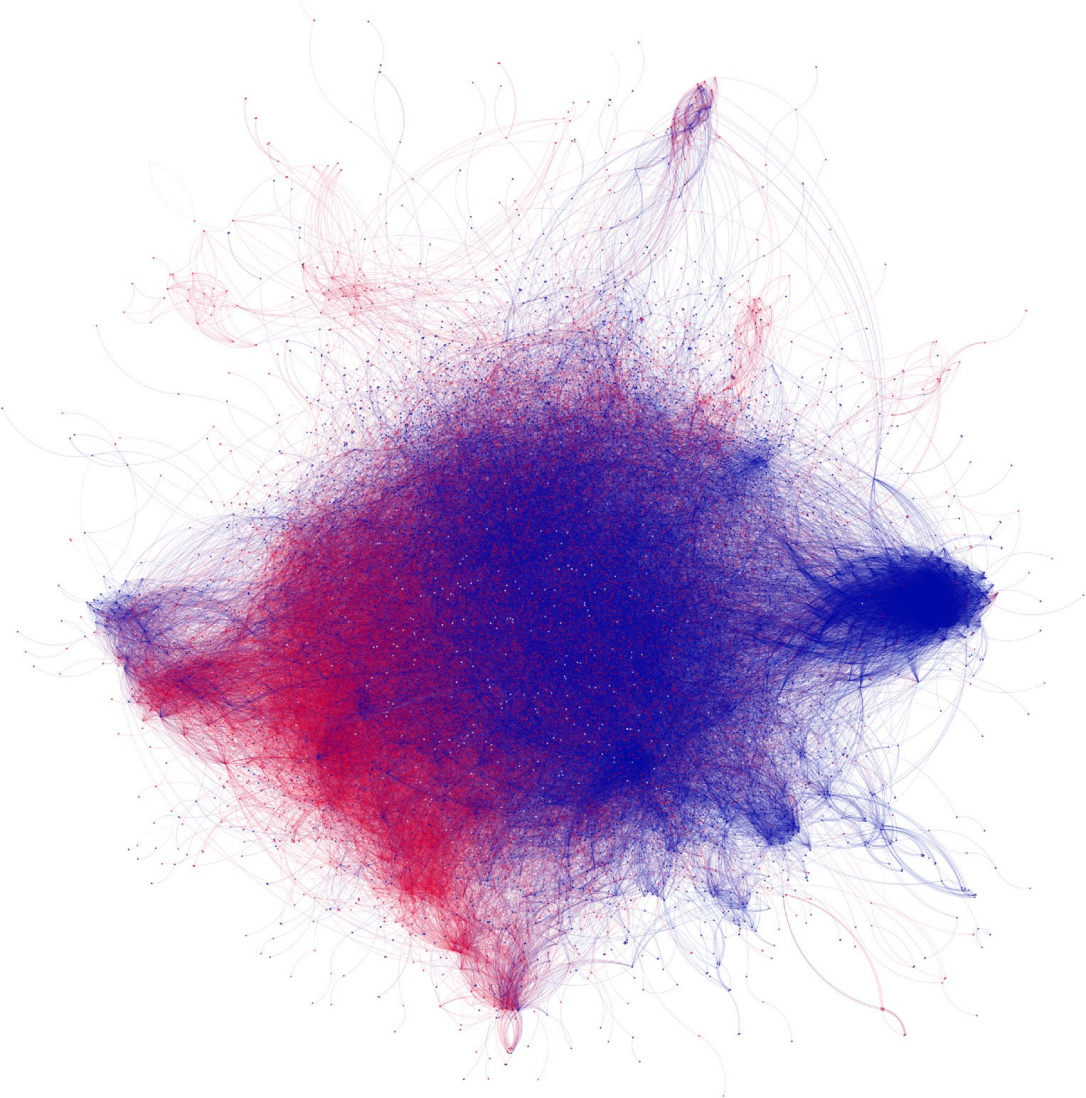
Reproducing partisan linguistic clusters even when explicitly partisan terms are removed from Twitter bios. The plot contains the 11,073 users who included explicit partisan terms in their Twitter bios: 54% who used exclusively or mostly liberal terms (blue), 42% who used exclusively or mostly conservative terms (red), and 4% who used an equal number of conservative and liberal terms (white). Each node is a user, with edge weights representing the cosine similarity between each pair of bios. (excluding explicitly partisan terms) in their bios (see Fig. 1 and Methods for more detail). The clear division into red and blue clusters shows that even in the absence of explicit partisan language, clear rhetorical boundaries emerge between liberal and conservative users. The modularity of the semantic network using these red and blue labels is .17, suggesting substantial partisan boundaries even in the absence of explicitly partisan language.

To answer this question, we employ a user network where each node represents a Twitter user, and the edge weights are based on the linguistic similarity between bios (see Fig. 1 and ’Methods” section for more detail on network construction). For this network, we only retain the 11,073 users who have at least one explicitly partisan term in their bio; this is only 1.8% of all users, reflecting that despite the variety of implicit partisan signals available in Twitter bios, the people who wish to explicitly highlight their partisanship remain relatively rare. Next, we remove all explicit partisan terms from their bios and plot the term co-usage network based *only* on the other (not explicitly partisan) bio terms.

Figure 3 plots this term co-usage network, with nodes colored blue and red corresponding to whether the user employed (exclusively or mostly) explicitly Democratic/liberal or Republican/conservative terms in their bios. In the co-usage network, users cluster consistently with their co-partisans, *even when the partisan terms themselves have been removed from their bios*. This indicates that usage of words implicitly signaling partisan identity—such as those examined in Fig. 2—is sufficiently consistent on its own to create clear linguistic divisions between liberal and conservative users.

These divisions are further evidenced in two formal tests. First, we calculated the modularity of the network given the red and blue labels shown in Fig. 3; the score of .17 indicates that semantic connections among users are about 18 percent greater than we would expect by random chance^39^. Second, we trained a random forest model to classify bios into Democratic/liberal or Republican/conservative categories without using any of the explicitly partisan terms in the bios. Using 80 percent of bios as training data, the model was able to correctly classify 74 percent of the bios in the remaining test set. Together, these analyses suggest that one would not need to see explicit partisan signifiers in order to infer most users’ partisan leanings with substantial (though not perfect) accuracy; the other terms they use to identify themselves would already be a strong enough signal.

## Discussion

Through an analysis of the terms that active Twitter users employ to describe themselves, we find that strong signals of partisan identity need not include any explicit reference to political ideology or a major political party. Instead, many seemingly politically neutral terms—like “artist,” “scientist,” or “christian”—are sufficiently aligned with political labels to serve as robust signifiers of one’s partisan identity. Taken together, these findings offer tangible evidence of cultural polarization and the politicization of lifestyle^12,14,17,40,41^.

Even if many of the common words that people use to describe themselves in Twitter bios have an identifiable partisan valence, individual users could hypothetically employ “cross-cutting” styles of self-presentation that mix liberal and conservative identifiers. In this way, users would resist easy partisan categorization. However, users who used explicitly partisan identifiers *also* tended toward similarity with their co-partisans with regard to the *other* words they used to describe themselves in realms outside of explicit partisan politics. As a result, it is possible to characterize the partisan identities of most users even if we were to ignore any explicitly partisan language in their bios. To use an offline analogy, imagine that you see a car driving down the street with bumper stickers advertising veganism and yoga. Even if the car does not have any sticker explicitly invoking partisan politics, you would likely correctly infer that the driver is liberal. Similar inferences are made possible online by the variety of partisan-coded signals one can see in the things that social media users choose to highlight when presenting themselves for their online audience.

By saying that seemingly non-partisan identities like “scientist” or “christian” *signal* partisan identity, we are not claiming any *causal* relationship among these elements. In many such instances, we would expect that the relationship between partisan and non-political identities might instead reflect a larger set of relationships involving yet other unmeasured elements of peoples’ sociodemographic background^12^. For example, the simplest explanation for the connection between “christian” and Republican/conservative labels lies in a joint connection to religious preference and religiosity, which may in turn also be connected to age, race, geographic region, urban/rural location, and any number of other parts of one’s background. However, the key point of our analysis is that—unlike in social science surveys where people respond to questions eliciting such sociodemographic information—the users in our data are not responding to any such prompts. Insofar as sociodemographic information is not systematically visible or obvious in an online setting, we might expect interactions on Twitter to be less subject to stereotypical inferences people might make in everyday interactions. However, our analysis shows how this might not be the case, as even many of the non-political labels people use to describe themselves in Twitter profiles are also generically correlated with partisan identity, and thereby allow for inferences about someone’s likely politics even in the absence of explicit statements to that effect.

In fact, the design of Twitter and other mass-usage social media platforms might promote partisan signaling and cultural polarization much more efficiently than would offline interactions. This is because of the demand that each user craft a single, immutable, non-context-dependent “identity” to show to the world. In offline interactions, people have greater ability to choose which identities to highlight and which others to hide or downplay depending on the social context and the “audience” with which they are interacting^30^. However, social media collapses different audiences into one—for example, one cannot present one Twitter bio to one set of followers and a different one to another set of followers^42,43^. As a result, users cannot selectively emphasize different identities in a way that might signal solidarity with one’s co-partisans but also downplay differences with opposite-partisans. The results of this study highlight how the non-context-dependent nature of self-presentation on Twitter might reinforce the social and cultural distance between people with differing political views.

Twitter bios provide large-scale behavioral data on styles of self-presentation that would not be measurable in a population survey. While active Twitter users are not representative of the population as a whole, Twitter itself is an important object of study in its own right as a context where social interactions amplify polarization^10,28,44,45^. Political narratives and partisan polarization on Twitter also spill beyond the platform itself, with many journalists being active Twitter users and news media often addressing political Twitter drama in their regular news coverage^46–49^. This study shows how social media can reflect and reinforce partisan divisions in ways that go beyond the polarizing effects of interactions with other users, extending even to the way that we describe and present ourselves on these platforms. Further research is needed to more fully understand what drives different styles of self-presentation on social media, and the role that these dynamics play more broadly in reflecting and amplifying polarization.

## Methods

### Sample

For computational feasibility, we randomly sampled 1 million active-user Twitter bios from a larger dataset of approximately 6 million bios. Data were collected through the Twitter API from 1% of all U.S.-based, publicly-available tweets in August 2019. Any user with at least one tweet in these data was included in the full list of bios, from which we then drew the random sample. However, users who tweet more frequently would be more likely to have at least one tweet in the 1% sample on the Twitter API, so that our sample should not be viewed as a random sample of all Twitter accounts, a population that would include many dormant and inactive users. According to a survey from Pew Research Center, the race and gender demographics of U.S. Twitter users are similar to the overall U.S. population, while age and political leanings do not reflect overall U.S. demographics, with Republicans and people over 50 being much less likely to use the platform^50^. These differences are noteworthy and require us to be cautious about generalizations beyond active Twitter users.

### Text cleaning

Of the 1 million Twitter bios sampled, 188,564 bios did not contain any text, and were therefore removed from the dataset prior to text cleaning. Standard text analysis preparation techniques were employed to clean the 811,436 Twitter bios that contained text data. First, all URLs (most of which referenced users’ other social media accounts) were removed from the bios. Second, we removed punctuation, numerals, symbols, emojis, and special characters. All characters were also made lowercase. Third, stopwords (e.g., “to”, “I”, “am”, “the”) were removed using existing stopwords dictionaries for English, Spanish, and Portuguese languages (the three most common languages in the dataset) through R’s “tm” package. Lastly, words were stemmed such that similar words like “computer”, “computation”, “computing”, “computational”, and “compute” would all be stemmed to the root “comput” and counted as the same term.

Given the sheer volume of unique words that appear across a large set of Twitter bios, it was also necessary to remove terms that did not occur with enough regularity to be meaningfully analyzed. To focus on commonly-used words, we removed terms that did not occur in at least 0.1% of the Twitter bios. After removing infrequently used terms, we proceeded to remove Twitter bios that had fewer than 2 words, as such bios would not generate any linkages between co-used terms. The removal of these empty and near-empty bios left us with a final sample of 677,132 Twitter bios.

### Partisan terms

Our list of explicit partisan terms used Rogers and Jones’^29^ previous list of terms as a foundation. However, we supplemented their list using additional criteria. Specifically, we consider “explicit” partisan terms to be unambiguously political and partisan in meaning. Many words or phrases have political connotations (e.g., “Black Lives Matter”) but are not explicitly tied to a political party, and were therefore not included in the category of explicit partisan terms (though with enough frequency, such terms could still be included in the overall term network).

**Figure 4:**
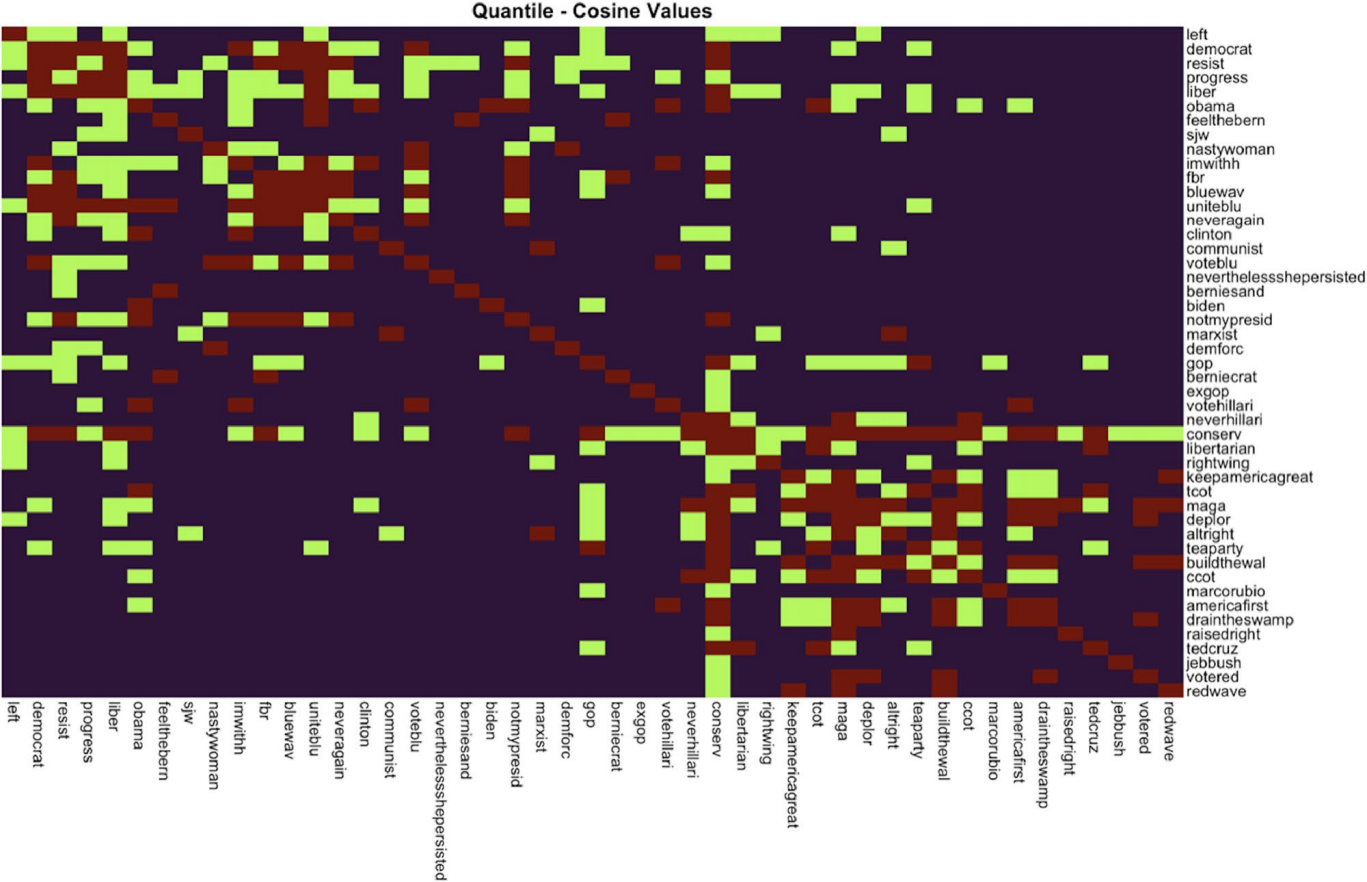
A heat map for quantile cosine values. Red cells indicate higher cosine similarity among terms, followed by green and then blue. Partisan terms are most similar to other co-partisan terms. Cosine similarity scores among Democratic/liberal terms appear in the top-left while scores among Republican/conservative terms appear in the bottom-right.

We defined explicitly partisan terms as those invoking (1) established partisan labels or ideological labels closely identified with either party (i.e., Republican, Democrat, liberal, conservative), (2) references to party leaders (e.g., Donald Trump, Hillary Clinton, Bernie Sanders, Ted Cruz, etc.), (3) partisan candidate campaign slogans and phrases (e.g., “feel the bern,” “make america great again,” “I’m with her,” etc.), (4) partisan voter phrases (e.g., “blue wave,” “vote red,” etc.), (5) party opposition phrases (e.g., “exgop,” “fight back resistance,” etc.), and (6) party-aligned ideological groups (e.g., “tea party,” “marxist,” “communist,” “libertarian”). In addition to stringent criteria, we tested how well these terms hang together in actual bios; Fig. 4 summarizes these patterns of co-occurrence with a heat map. Then, the first author examined each Twitter bio with at least one partisan term to see if other partisan terms should be included based on our criteria. Lastly, we examined all 1,604 network terms to ensure that no other frequently-used explicitly partisan terms were missed.

In cases where multiple words work together to indicate explicit support for a party or party leader for example, the phrase “make america great again”), these were collapsed into single terms. This had the added benefit of matching hashtags of the same phrases. We then aggregated all explicitly Republican and conservative/right-wing labels under the umbrella term “conservative”, and did the same for Democratic and liberal/left-wing labels under the term “liberal” (see Supplementary Materials for a complete list of explicit partisan terms). This binary labeling reflects the contemporary context where political ideology (i.e., liberal or conservative) and party identification (i.e., Democrat or Republican) have become much more closely identified than in the past when there were more conservative Democrats and liberal Republicans than now.

### Measures

In this study, we aim to understand non-political aspects of culture or lifestyle that are imbued with partisan meaning. In studying Twitter bios, we are able to examine this relationship using two unique approaches: examining the relations between terms based on co-occurrence in bios (term network) as well as the relations between users based on term co-usage (user network).

#### Term network

For the first approach, we consider individual terms in Twitter bios as the key units of interest. The corpus of Twitter bios was transformed into a document-term matrix, where the cell values were weighted using *term frequency-inverse document frequency*. *Term frequency* (TF) is the number of times a given term appears in a document compared to the total number of words in the document:

$$\begin{aligned} TF = \frac{\text {number of times the term appears in the document} }{ \text {total number of terms in the document}}. \end{aligned}$$

The *inverse document frequency* (IDF) represents the proportion of documents in the corpus that contain the term. Words unique to a small percentage of documents (e.g., liberal, conservative, Republican, Democrat) receive higher importance values than words common across many documents (e.g., love, like, follow):

$$\begin{aligned} IDF = log (\frac{\text {number of documents in the corpus}}{\text {number of documents in the corpus containing the term}}). \end{aligned}$$

The *term frequency-inverse document frequency* (TF-IDF) of each cell in the document-term matrix is then calculated by multiplying TF and IDF scores, weighting the appearance and distinctiveness of each term relative to the document length. In other words, the importance of a term in a given document is high when it occurs frequently in a given document and rarely in others:

$$\begin{aligned} \textit{TF-IDF} = TF\cdot IDF. \end{aligned}$$

The TF-IDF-weighted matrix is then transformed into a term-by-term cosine similarity matrix that reflects semantic similarity, such that pairs of terms with high similarity are those that disproportionately appear in the same bios. From this cosine similarity matrix, we create a network object using the R package “igraph.” In this network, nodes are individual terms and each edge is weighted to represent the cosine similarity between terms (see Panel A of Fig. 2) or

$$\begin{aligned} \cos (\textbf{x},\textbf{y})= {\mathbf{x \cdot } \textbf{y} \over \Vert \textbf{x}\Vert \Vert \textbf{y}\Vert } = \frac{ \sum _{i=1}^{n}{\textbf{x}_i\textbf{y}_i} }{ \sqrt{\sum _{i=1}^{n}{(\textbf{x}_i)^2}} \sqrt{\sum _{i=1}^{n}{(\textbf{y}_i)^2}} } \end{aligned}$$

where *x* and *y* are pairs of terms represented by vectors, where each entry in a vector is a TF-IDF score of the term in a document, and *n* is the number of documents in the corpus. To ease computational burdens and figure production, a minimum cosine value threshold was set to remove the weakest ties ($<.02$) from the network.

#### Term partisan scores

A term’s network edge-weight (strength of the weighted tie) with each of the aggregated nodes containing explicitly partisan liberal and conservative terms, respectively, was used to create both a *liberal cosine score* and a *conservative cosine score* for that term. Since these edge weights already reflect TF-IDF adjustments and transformation into cosine similarities, a term that receives a high liberal score, for example, would be one that disproportionately tended to co-occur in bios that also employed explicitly liberal terms.

#### User network

For the final part of our analysis, we turned our focus to network representations of semantic linkages among active Twitter users themselves (see Fig. 3). More specifically, we were interested in the non-political network structure of the Twitter bios that we knew to have a partisan lean. To distinguish the partisan lean of bios, we produced a liberal count score and a conservative count score for each user by summing the total number of explicit liberal and conservative terms used in each Twitter bio. Bios that did not include at least one liberal or conservative term were then excluded, so only bios with a clear partisan lean would be included in the network. *User partisan scores* were then calculated for each Twitter bio by subtracting the bio’s conservative score from the bio’s liberal score, such that a positive *user partisan score* indicates that the bio contained more explicitly liberal terms, while a negative score suggested it was more conservative. A score of zero indicated that the bio contained an equal number of liberal and conservative terms, and we could not infer its partisan lean.

To create the user network, we used textstat_simil command from the quanteda.textstat R package to project a cosine similarity matrix of documents (i.e., user bios) from the document term matrix of the partisan bios. The textstat_simil computes a cosine similarity score between each pair of documents based on the terms within each document, such that documents with more similar language (relative to term frequency across all documents) have a higher cosine similarity. Using this cosine matrix, we produced a network of Twitter bios, in which each node represented a user, and each weighted network tie represented the cosine similarity value between those two documents. As with the term network, we included a minimum cosine threshold that removed the weakest ties ($<0.2$) in the network. The difference in minimum thresholds between the two networks reflects the differences in typical cosine values between the term and user networks. After removing edges that did not meet the threshold, we assigned equal weights to all remaining edges; however, results were virtually identical (with a slightly higher modularity of 0.19 compared to 0.17 in the binarized network) when edges above the threshold retained different weights. Once the user cosine network was created, the *user partisan scores* were aggregated as a node attribute so that nodes could be colored by partisan lean.

### Supplementary Information

Supplementary Table S1.

## Data Availability

While raw Twitter data cannot be openly published due to Twitter’s Terms of Service, derivative data (i.e., document frequency matrices and cosine similarity data) used to conduct these analyses have been uploaded to the Open Science Framework’s public data repository (https://osf.io/e6v3h/?view_only=8fb2f684aa164028b6a6157cd0f3492b).

## Code availability

Code used to calculate statistical analyses and produce both term and partisan networks are provided as part of the replication package, which is available at the Open Science Framework’s repository (https://osf.io/e6v3h/?view_only=8fb2f684aa164028b6a6157cd0f3492b).
