# Supplementary material for: Partisan styles of self-presentation in U.S. Twitter bios
Source: Sci Rep. 2024 Jan 11;14:1077. doi: 10.1038/s41598-023-50810-0 (PMC10784547; doi:10.1038/s41598-023-50810-0)
Supplement: Supplementary file 1 — Supplementary Table S1. [file 41598_2023_50810_MOESM1_ESM.pdf]

Supplementary Materials for Partisan styles of self-presentation in U.S. Twitter bios

Liam Essig[1,2*] and Daniel DellaPosta[1,2]

[1]Sociology & Criminology, Pennsylvania State University, Street, University Park, 16802, PA, USA.
[2]Social Data Analytics, Pennsylvania State University, Street, University Park, 16802, PA, USA.

*Corresponding author(s). E-mail(s): lwe5065@psu.edu;
Contributing authors: djd76@psu.edu@;

Table S 1 Comprehensive list of explicitly partisan terms used to compute the aggregated "Conservative" and "Liberal" terms.

Explicitly Partisan Terms	
Conservative Terms	Liberal Terms
altright	berniecrat
americafirst	bernie sanders
buildthewall	joe biden
ccot	bluewave
conserv	hillary clinton
deplorable	communist
drainttheswamp	dem
gop	demforce
jeb bush	democrat
keepamericagreat	exgop
libertarian	fightbackresistance
makeamericagreatagain	feelthebern
marco rubio	imwithher
neverhillary	left
pence	liber
raised right	marxist
red wave	nastywoman
republican	neveragain
rightwing	neverthelessshepersisted
reagan	not my president
tcot	obama
teaparty	progress
ted cruz	resist
donald trump	sjw
votered	takeaction
	uniteblue
	votebernie
	voteblue
	votehillary

Note: The list of explicit terms includes several partisan acronyms. For conservatives, these include "conservative christians on twitter" (ccot) and "top conservatives on Twitter" (tcot). For liberals, this includes "fight back resistance" (fbr) and "social justice warrior (sjw). Other words or phrases are references to partisan discourse, including words intended to offend members of the opposite party but were embraced by their opposition. For example, many conservative Twitter bios began including "deplorable" or "proud deplorable", a reference to Hillary Clinton calling Trump supporters a "basket of deplorables". Similarly, liberal accounts reclaimed the phrase "nasty woman" that Trump had used to insult Hillary Clinton. "Nevertheless, she persisted" was embraced by liberals in 2017 when Senator Mitch McConnell used the phrase when he criticized Senator Elizabeth Warren.
